# Supplementary material for: Inappropriate timing of salt intake increases the risk of heat-related illness: An observational study
Source: PLoS One. 2024 Jan 2;19(1):e0296388. doi: 10.1371/journal.pone.0296388 (PMC10760686; doi:10.1371/journal.pone.0296388)
Supplement: Attachment — Submitted filename: Response_to_reviewer 20230425.docx [file pone.0296388.s001.docx]

Response to reviewer

Dear Reviewers,

We appreciate for your constructive comments on our paper. We have carefully revised the manuscript text based on your comments. The point to point responses are below.

Reviewer #1: Thank you very much for the opportunity to review this very interesting paper.

However, I believe that two main points need to be fundamentally modified

(1) Sample size

The main analysis is a multivariate logistic analysis with 7 covariates, but you have not considered whether the sample size of 28 participants is sufficient for statistical analysis.

I apologize if the number of participants is incorrect. However, even in that case, it is difficult to understand the attributes of the participants, and we would appreciate clarification.

*Response: To clarify, there were 28 study participants. In total, they recorded outdoor training for 250 days. The sample size sufficient for statistical analysis was 250 working days. In the Methods section of the revised manuscript, we have mentioned the total sample size.*

2) Attributes of each group in the Salt intake

Since the details of each of the groups are not clear, it is not possible to determine whether Salt intake can be considered a significant factor.

*Response: We apologize for this lack of explanation. We have added this detail in the revised manuscript.*

I am sure there are other points to consider, but if you are going to submit a revised submission, I would appreciate it if you could respond to the above two points as soon as possible and then review it again.

*Response: Thank you for your comments. We have carefully reviewed your comments and addressed them in the revised manuscript.*

Reviewer #2: 1. Specific errors here:

37 line: indiacte

73 line: minimze

84-85 line: salt intake in HRI aims

89 line: emphasizing

102: in accordance with

239: may actually

271: a daily basis

*Response: We apologize for these typographical errors. We have corrected them in the revised manuscript. In addition, we have had the manuscript checked by a native English speaker from a professional editing company to ensure that there are no remaining grammatical or syntax errors. We hope that the revised manuscript is now suitable for publication.*

2. Material and Methods /

2.1. Study design and participants.

Please include inclusion criteria, heat acclimation criteria. Describe the selected Fukushima Prefectural Fire Department sites, total number of possible participants. Add in the annexes the Informed Consent and the Approval of the Ethics Committee.

*Response: In the revised manuscript, we have detailed the inclusion criteria, heat acclimation criteria, selected fire department, total number of fire departments, and recruited subjects. We have also submitted the annexes you have mentioned to the editorial office.*

2.3 Data Collection.

Please add in the annexes the questionnaires before and after the training, also clarify how they took the value during the training.

*Response: The original version of the questionnaire is written in Japanese. In this resubmission, we have submitted the questionnaire as an annexed file. Participants administered to questionnaire after the training.*

Define the variables contained between lines 111 to 114. Define the parameters under which HRI was defined. How the salt tablets were prepared and how they were administered.

*Response: As per your comment, we have clarified the definition of HRI in the Statistical analysis section . Details regarding the salt tablets are described in line 131.*

Define how weight and height measurements were taken, what equipment was used.

*Response: Data on height were obtained from health checkup records. Body weight was measured using Body Composition Analyzer BS-230 (Dretech Co., Ltd, Saitama, Japan). We added these details in the revised manuscript (lines 127–128).*

What information was taken from the medical history.

*Response: From the medical history, we collected information on the history of hypertension and diabetes mellitus because these diseases increase the risk of HRI.*

Add the BDHQ questionnaire, which method was used to calculate alcohol consumption, number of responses not given.

*Response: We cannot provide the BDHQ as it is a copyrighted material. We have instead included a reference paper demonstrating the reliability of the BDHQ in nutritional surveys.*

Were the dietitians previously trained?

*Response: Yes, one dietitian (co-author TI) was trained to evaluate the BDHQ.*

Were the weather conditions evaluated with those taken at the site and their relationship with HRI?

*Response: Yes, daily maximum WBGT was included as a confounding factor, and it indicated a negative result.*

3. Results.

How did you calculate the concentration of salt in the diet and if you took salt tablets, how and at what time?

*Response: The concentration of salt in the diet was estimated from the BDHQ. The BDHQ was administered in the beginning of July.*

It would be possible to add more sociodemographic information by site, including gender, to Table 1.

*Response: All firefighters in the study department were men. Since one participant trained at multiple sites, the demographic data for each site could not be described.*

What time of day did the training take place?

*Response: The fire department scheduled training time twice daily (morning and afternoon), and all firefighter engaged training once in a day without emergency dispatch.*

In table 3, can the information be disaggregated by each site?

*Response: We have added more information in Table 3 and corrected a typographical error. However, this error did not affect the results of logistic regression analysis.*

How was the information collected during the training?

*Response: As described in lines 118-119, study participants answered a self-administered questionnaire before and after training.*

4. Conclusion

It is suggested to support the conclusions with part of the information placed in the summary.

*Response: Yes, we have revised the conclusions section and unified the content with that in the Abstract.*

5. Additional comments for the author.

Personally, I consider it to be excellent research whose results will help improve the treatment and prevention of HRI.

I believe that the research has much more data to contribute, which were taken during the field work, it is possible that its analysis and a more robust correlation of the variables could increase the conclusions, achieving greater indications for the prevention of this pathology.

*Response: Thank you for your favorable review of our manuscript. We have carefully revised our paper to sufficiently address all your comments.*
