# Supplementary material for: Inappropriate timing of salt intake increases the risk of heat-related illness: An observational study
Source: PLoS One. 2024 Jan 2;19(1):e0296388. doi: 10.1371/journal.pone.0296388 (PMC10760686; doi:10.1371/journal.pone.0296388)
Supplement: Attachment — Submitted filename: Response_to_reviewer 0821.docx [file pone.0296388.s002.docx]

Response to the reviewer

Dear Reviewers,

We appreciate your constructive comments on our paper. We have carefully revised the manuscript text based on your comments. Our point-to-point responses are below.

Reviewer #1: The description of the statistical analysis is still confusing.

Regarding the sample size, which we pointed out in the last issue, the process of calculating the required number of 250 cases is not clear.

However, if I were to accept your assertion, then 28 participants would require 250 cases, which would mean multiple data collections from a single patient.

From the statement "Table 4. logistic regression analysis of heat-related illness risk.", I assume that a multivariate logistic analysis was performed.

However, in principle, in a multivariate logistic analysis, the outcome is measured once per patient.

From your description, I can only follow the above inference. From this inference, I cannot say that you have performed the statistical analysis correctly, and I have no choice but to reject your paper.

*Response: We used logistic mixed effect model instead of logistic regression model. We have revised the description related to Table 4 following the results of the recalculations.*
