# Supplementary material for: Inappropriate timing of salt intake increases the risk of heat-related illness: An observational study
Source: PLoS One. 2024 Jan 2;19(1):e0296388. doi: 10.1371/journal.pone.0296388 (PMC10760686; doi:10.1371/journal.pone.0296388)
Supplement: Attachment — Submitted filename: Response_to_reviewer_1211.docx [file pone.0296388.s003.docx]

Response to the reviewer

Dear Editor,

We appreciate your constructive comments on our paper. We have carefully revised the manuscript text based on your comments. We explained why we selected mixed-effect model in the response for reviewer #1.

1. Some supporting citation(s) for the new statistical approach adopted.

*Response: Thank you for your suggestion. We added references that support our new statistical method (citation No28 and 29).*

2. Limitations of the study, clearly indicating that the results reported should be treated with caution considering the volume of the data generated in the study.

*Response: We have noted the need for caution regarding the handling of the results in the limitation section.*

3. Directions for future research under CONCLUSIONS.

*Response: We added sentence in conclusions section.*

Dear Reviewers,

We appreciate your constructive comments on our paper. We have carefully revised the manuscript text based on your comments. Our point-to-point responses are below.

Reviewer #1: I addressed the questions regarding logistic analysis and study design in the previous peer review.

I had expected that you would revise the study design, but you have utilized a different statistical technique.

There was no response to my comments on what led to the use of this statistical technique.

Since I am not a statistician, I cannot judge the validity of the conclusions you have drawn from the results of this analysis without comment.

I have assigned a Major Revision for this peer review.

If the manuscript is to be resubmitted, it needs to be evaluated by reviewers other than me.

***Response****: The mixed-effects model is a statistical method used in studies where the same individual is measured repeatedly, such as in the current study. Information that can identify individuals and information that takes the same value for multiple subjects are referred to as fixed effects, while information that varies by measurement is treated as random effects and incorporated as variables. We have added a citation for a paper that utilizes these mixed-effects models.*
